# High-Accuracy Height-Independent 3D VLP Based on Received Signal Strength Ratio

**DOI:** 10.3390/s22197165

**Published:** 2022-09-21

**Authors:** Yihuai Xu, Xin Hu, Yimao Sun, Yanbing Yang, Lei Zhang, Xiong Deng, Liangyin Chen

**Affiliations:** 1 College of Computer Science, Sichuan University, Chengdu 610065, China; 2 Center for Information Photonics and Communications, Southwest Jiaotong University, Chengdu 611756, China; 3 Institute for Industrial Internet Research, Sichuan University, Chengdu 610065, China; 4 School of Information Science and Technology, Southwest Jiaotong University, Chengdu 611756, China

**Keywords:** visible light positioning, indoor position system, received signal strength, high accuracy, weighted least squares

## Abstract

Visible light positioning (VLP) has attracted intensive attention from both academic and industrial communities thanks to its high accuracy, immunity to electromagnetic interference, and low deployment cost. In general, the receiver in a VLP system determines its own position by exploring the received signal strength (RSS) from the transmitter according to a pre-built RSS attenuation model. In such model-based methods, the LED’s emission power and the receiver’s height are usually required known and constant parameters to obtain reasonable positioning accuracy. However, the LED’s emission power is normally time-varying due to the fact that the LED’s optical output power is prone to changing with the LED’s temperature, and the receiver’s height is random in a realistic application scenario. To this end, we propose a height-independent three-dimensional (3D) VLP scheme based on the RSS ratio (RSSR), rather than only using RSS. Unlike existing RSS-based VLP methods, our method is able to independently find the horizontal coordinate, i.e., two-dimensional (2D) position, without a priori height information of the receiver, and also avoids the negative effect caused by fluctuation of the LED’s emission power. Moreover, we can further infer the height of the receiver to achieve three-dimensional (3D) positioning by iterating the 2D results back into positioning equations. To quickly verify the proposed scheme, we conduct theoretical analysis with mathematical proof and experimental results with real data, which confirm that the proposed scheme can achieve high position accuracy without known information of the receiver’s height and LED’s emission power. We also implement a VLP prototype with five LED transmitters, and experimental results show that the proposed scheme can achieve very low average errors of 2.73 cm in 2D and 7.20 cm in 3D.

## 1. Introduction

The development of the Internet of Things (IoT) is facilitating a rapid increase in the number of automated robots and transporters used in factory warehouses and indoor public places [1,2]. One of the basic requirements of these devices is to know their own locations. Traditional methods of indoor positioning are based on radio frequency (RF) technologies such as WiFi [3,4] and Bluetooth [5]. However, they have some weaknesses, such as high power consumption, congested channel interference, and low accuracy. As a developed classic technology, sound-based positioning is also used in some large indoor scenes (e.g., greenhouses) and provides better accuracy than RF-based positioning [6,7], but it generates noise and requires a low level of environmental noise. SLAM-based positioning systems have also been applied to indoor positioning [8]; however, they are normally expensive because of the use of specialized equipment, such as laser sensors, to scan the surroundings and construct the map. In recent years, the widespread use of light emitting diode (LED) lights has promoted visible light communication (VLC) technology, which also supports positioning. The LED-based positioning is also known as visible light positioning (VLP), which is considered a potential indoor positioning technology thanks to its advantages of high accuracy, low deployment cost, weak multipath effect, immunity to electromagnetic interference, and sufficient channel resources [9,10].

A VLP system generally consists of multiple LED transmitters (also known as anchors) and a receiver. A photodiode (PD) receiver explores the received signal strength (RSS) from multiple LED transmitters, normally more than three transmitters, to locate its own position. To be more detailed, the distance between the receiver and one transmitter can be inferred from the RSS given the known emission power of the LED transmitter and light propagation model [11]. By further combining the multi-distances from the other transmitters, we can finally position the receiver with trilateration. Moreover, there are some other ranging methods, such as time difference of arrival (TDOA) [12] and time of arrival (TOA) [13] for localization. However, TDOA requires clock synchronization of either the transmitter or the receiver, while TOA requires clock synchronization of both, which is hardly realized in a practical low-cost VLP system. Although the angle-of-arrival (AOA) [14] localization has no such requirement of clock synchronization, it typically needs a sensor array consisting of multiple PDs, cameras, or other hardware at the receiver side, which is also not suitable for very low-cost IoT applications. Additionally, the VLP system can also be achieved by using an image sensor (e.g., CMOS) as a receiver to derive the distance using the ratio of the LED size to its projection size [15,16,17,18], but cameras are normally high-cost and power-intensive and not suitable for low-power scenarios. Compared to the above-mentioned methods, RSS-based positioning is preferred in VLP since the RSS is easy to be measured with low-power and low-cost hardware.

Current research attention towards RSS-based positioning is devoted to improving accuracy and robustness by introducing more hardware. The study in [19] applies two kinds of LEDs in a single lamp to deal with the performance loss in multi-path propagation scenes. The proposed DM-LED system has been validated through both simulation and experimental results. The work of [20] equips two PDs in a receiver to avoid the ambient light noise by transforming the ratio of RSS to the incidence angles of two PDs, where the proposed DarkVLP system has been corroborated by experimental results. The channel state information (CSI) was considered in [21] to extract the received power of the line-of-sight (LoS) signal to reduce interference from the reflected paths so that the accuracy loss of triangulation can be alleviated. However, the practicability is uncertain since there is only simulation validation. The model-based methods described above have achieved significant results, but most of them have additional requirements for a priori information, e.g., object height, rotation angle, and LED emission power, which should be known and constant. In practical applications, these a priori parameters are usually difficult to obtain and are varying, especially the relative height of the receiver. In large buildings, e.g., warehouses and factories, the floor is not always horizontal at the same height, and the relative height of the receiving device may be changing with its movement, which makes these methods not applicable.

The prosperity of machine learning (ML) provides many data-driven methods to solve nonlinear problems, including VLP. The methods based on regression [22], reinforcement learning [23], and neural networks (NNs) [24,25] typically obtain higher accuracy and stability than the traditional ones. However, they require offline progress to collect data sets or fingerprints to train models. Once the environment or the system changes, the network probably provides poor positioning estimation. As the running time increases, these changes are inevitable, so the accuracy loss cannot be avoided. In such a case, new data need to be collected, and the model should be trained again. Although researchers are dedicated to reducing the amount of data collection [26,27], ML-based methods are still highly time-consuming and have poor environmental stability compared to their model-based counterparts.

This paper focuses on removing the dependence on height in the VLP system and developing a high-accuracy positioning method. The RSS measurements are transmitted to the ration (RSSR) in preprocessing so that the emission power is unnecessary. An accurate height-independent positioning method based on RSSR is proposed, which solves horizontal coordinates without the a priori information of the vertical, relative height between transmitter and receiver. Then, the vertical coordinate can be estimated after putting the horizontal estimation back into the original equation, composing a three-dimensional (3D) position estimator of the target receiver. Due to this feature, the proposed method is capable of achieving high accuracy. We construct a test platform in a 1.5 m × 1.5 m × 2.7 m space with five LEDs and one PD to evaluate the performance of the prototype. The proposed method achieves a mean error of 2.73 cm on the horizontal plane and 7.20 cm in 3D. Comparing the horizontal localization performance, the proposed method has better accuracy than that of the trilateral positioning [28] designed for 2D only and using the a priori height of the receiver and emission power of transmitters. We also compare the proposed method with the successive linear least squares (SLLS)-based method [29] and the ML-based method [22] for 3D localization when the receiver height and LED emission power are unknown. The proposed method outperforms both the SLLS-based and the ML-based methods.

The rest of the paper is organized as follows. Section 2 introduces the background of the classical 2D VLP method and points out the problems of its generalization to 3D VLP from a mathematical point of view. Section 3 presents the proposed system’s architecture and method. Section 4 evaluates the performance of the proposed method by designing experiments and comparing it with existing methods. Finally, Section 5 concludes this paper.

## 2. Indoor Positioning with Visible Light

**Figure 1:**
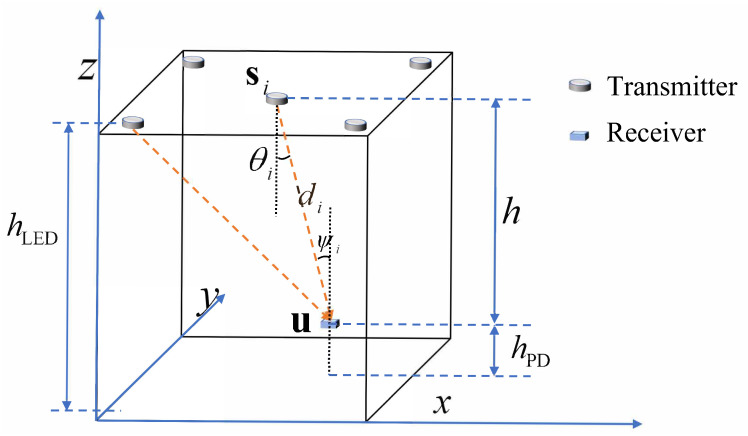
The basic model of VLP.

The layout of a basic RSS-based VLP system is shown in Figure 1. The system consists of *M* LED transmitters that are deployed on the ceiling with the same height $h_{\mathrm{LED}}$. The horizontal coordinates of these LED transmitters are denoted by $s_{i}={\left(x_{i},y_{i}\right)}^{T}\left(i=1,\cdots ,M\right)$. Assume that the PD of the receiver is placed vertically. It can convert light signals to electrical signals whose strength is proportional to the RSS. The unknown PD receiver has the horizontal coordinate $u={\left(x_{u},y_{u}\right)}^{T}$ and height $h_{\mathrm{PD}}$. Thus, the relative height of these transmitters to the receiver is denoted as $h=h_{\mathrm{LED}}-h_{\mathrm{PD}}$. The distances between the transmitters and the receiver are denoted by $d_{i}\left(i=1,\cdots ,M\right)$.

In this section, assuming that the value of RSS has been extracted in some way (e.g., frequency or time domain), according to the Lambert model, the RSS from the *i*-th LED is [11]

(1) $$\begin{array}{c} \begin{array}{c} P_{i}=\frac{(n+1)A}{2\pi {\left(d_{i}\right)}^{2}}\cos^{n}\left(\theta_{i}\right)\cos\left(\varphi_{i}\right)P_{0},\left(i=1,\cdots ,5\right) \end{array} \end{array}$$

where *A* is the receiving area of PD, $\theta_{i}$ is the radiation angle of the *i*-th LED, $\varphi_{i}$ is the incident angle between the PD and the ${\mathrm{LED}}_{i}$, and $P_{0}$ is the emitted signal strength of the LED. *n* is the Lambert order that describes the divergence of the LED light, which is given by $n=\frac{-\ln\left(2\right)}{\ln\left(\cos\left(\phi_{1/2}\right)\right)}$, where $\phi_{1/2}$ represents the semi-power angle of LEDs. Since the normals of LEDs are parallel to that of the PD, this makes $\theta_{i}$ equal to $\varphi_{i}$. Thus, (1) can be simplified as

(2) $$\begin{array}{c} \begin{array}{c} P_{i}=\frac{(n+1)A}{2\pi {\left(d_{i}\right)}^{2}}\cos^{n+1}\left(\theta_{i}\right)P_{0}=\frac{\left(n+1\right)AP_{0}h^{n+1}}{2\pi }\frac{1}{{\left(d_{i}\right)}^{n+3}},\left(i=1,\cdots ,M\right). \end{array} \end{array}$$

The parameters *A* and $P_{0}$ related to the LEDs and the PD can be measured in advance, and, in 2D VLP applications, the relative height *h* is also known as a constant so that $d_{i}$ can be determined as

(3) $$\begin{array}{c} \begin{array}{c} d_{i}=\sqrt[{n+3}]{\frac{\left(n+1\right)AP_{0}h^{n+1}}{2\pi P_{i}}},\left(i=1,\cdots ,M\right). \end{array} \end{array}$$

The distance $d_{i}$ can also be given by the geometric relationship between the *i*-th transmitter and the receiver so that the horizontal coordinates can be expressed by the following system of equations:

(4) $$\begin{array}{c} \begin{array}{c} \left\{\begin{array}{c} {\left(x_{u}-x_{1}\right)}^{2}+{\left(y_{u}-y_{1}\right)}^{2}+h^{2}=d_{1}^{2}\\ {\left(x_{u}-x_{2}\right)}^{2}+{\left(y_{u}-y_{2}\right)}^{2}+h^{2}=d_{2}^{2}\\ \vdots \\ {\left(x_{u}-x_{M}\right)}^{2}+{\left(y_{u}-y_{M}\right)}^{2}+h^{2}=d_{M}^{2} \end{array}\right.. \end{array} \end{array}$$

To ensure a unique solution, the number of equations cannot be less than three. Applying the least squares (LS) method to the system of equations, the horizontal coordinates of the receiver can be calculated as

(5) $$\begin{array}{c} \begin{array}{c} u={\left(C^{T}C\right)}^{-1}C^{T}D \end{array} \end{array}$$

where

(6) $$C=\left[\begin{array}{c} x_{2}-x_{1}\;\;\;\;y_{2}-y_{1}\\ \vdots \;\;\;\;\;\;\;\;\;\;\;\;\;\;\;\;\;\vdots \\ x_{M}-x_{1}\;\;\;\;y_{M}-y_{1} \end{array}\right],D=\frac{1}{2}\left[\begin{array}{c} \left(d_{1}^{2}-d_{2}^{2}\right)+\left(x_{2}^{2}+y_{2}^{2}\right)-\left(x_{1}^{2}+y_{1}^{2}\right)\\ \vdots \\ \left(d_{1}^{2}-d_{M}^{2}\right)+\left(x_{M}^{2}+y_{M}^{2}\right)-\left(x_{1}^{2}+y_{1}^{2}\right) \end{array}\right].$$

This is a proven, low-complexity method that has good performance when the RSS is interfered with by a low non-line-of-sight (NLoS) signal. However, it can be concluded from (3) that the method requires the relative height and the emission power as a priori information. As mentioned in Section 1, the height of the receiver is unknown in 3D positioning and the emission power varies with the environment (e.g., voltage fluctuations, temperature, and LED aging), which makes it challenging to generalize the trilateral localization designed for 2D scenes to 3D.

## 3. Height-Independent 3D VLP Enabled by RSSR

### 3.1. System Overview

**Figure 2:**
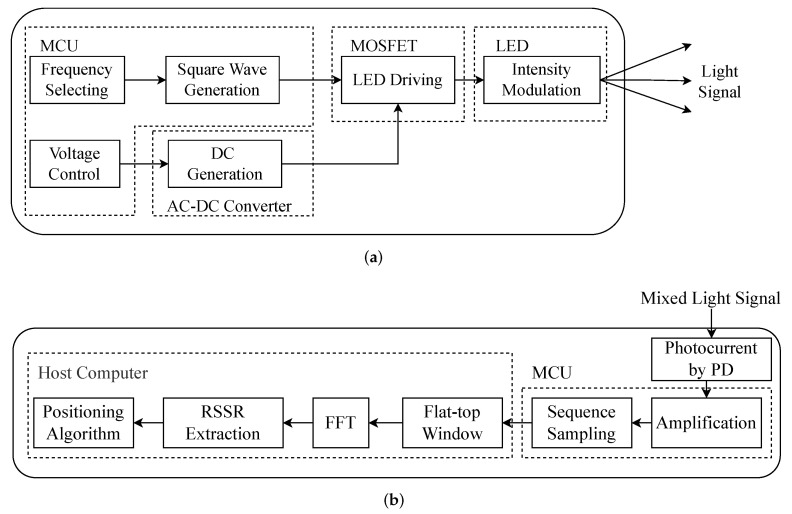
The diagrams of the main parts of the proposed VLP system: (**a**) low-cost MCU-based transmitter, (**b**) PD-based receiver.

As illustrated in Figure 1, the proposed system also consists of two main parts: transmitter and receiver. The transmitter modulates the LEDs and embeds the position information of the LEDs in different frequencies. As for the receiver, it is designed for receiving the light signals through a PD and positioning by processing the received signals. To address the challenges in Section 1 and Section 2, we equip the receiver with the proposed method, which mathematically circumvents the influence of the unknown height on the 2D positioning, and also performs 3D positioning in the absence of a priori information of the vertical plane. The workflow block diagram of the proposed system is shown in Figure 2, and the following subsections elaborate on the details one by one.

### 3.2. VLP Transmitter

**Figure 3:**
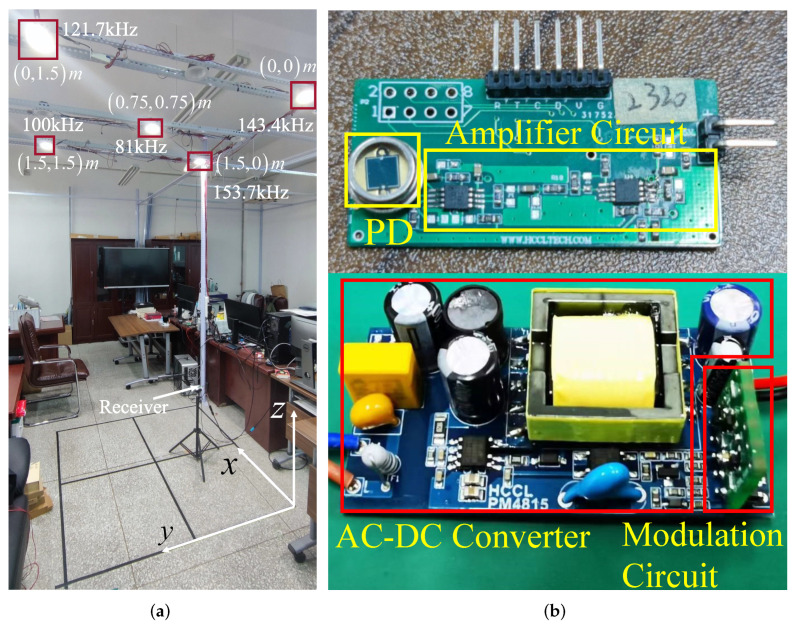
The experimental setup. (**a**) The experimental layout, and (**b**) the hardware design.

The transmitter consists of an AC-DC converter, a modulation circuit, and an LED light. Its real object is shown in Figure 3b. The modulation circuit consists of the microcontroller and its peripheral circuit. The workflow diagrams of the transmitter are shown in Figure 2a. The alternating current (AC) is sent into an AC-DC converter to obtain a stable direct current (DC), which can be further converted to square waves of different frequencies by generating a PWM wave to control the MOSFET in the peripheral circuit. Considering the requirement to separate the light signal from the frequency domain, and in order to avoid the harmonic frequencies of square waves interfering with each other, for the selected frequencies, it should be ensured that the harmonic frequencies are kept at a certain distance from the remaining main frequencies. Meanwhile, the VLC-based VLP system needs to satisfy the requirement of high-speed communication, so the frequencies must not be at a low level. According to the above constraints, combined with the microcontroller’s own clock frequency, the frequencies of the five LEDs are empirically set as 81 kHz, 100 kHz, 121.7 kHz, 143.4 kHz, and 153.7 kHz. In addition, to avoid saturation of the PD by excessive light from multiple LEDs, the voltage of the LEDs should be properly limited. We design a flyback converter to limit the voltage by low-frequency PWM before the DC passes through the MOSFET, which in turn controls the amplitude of the modulated signals.

### 3.3. VLP Receiver

The receiver is designed for sensing the light signals and processing the sampled data for extracting RSS to positioning. Specifically, the receiver consists of a PD, an amplifier circuit, and a microcontroller. As Figure 2b shows, the PD converts the light signal to the tiny current signal, which is further transferred by an amplifier circuit into the voltage signal capable of driving the analog-to-digital (ADC) of the microcontroller. Since the memory capacity of the microcontroller is limited, and to facilitate the experimental analysis, the sampled data are forwarded to the computer via a serial port. We consider real-time positioning on the microcontroller as future work.

The RSS is extracted by applying the fast Fourier transform (FFT) on the sampled sequence, and its accuracy is influenced by both the real frequency of the received signal and the frequency resolution. Some existing experiments [19,23] use a function generator to modulate the LED, while, in practical applications, a low-cost MCU is used to generate a frequency signal for modulation, which has difficulty in keeping the frequency stable. Moreover, the high frequency of the VLC system signal and the limited sampling window make the frequency resolution poor, meaning that the peak amplitude will leak to both sides if the main frequency of the signal cannot satisfy an integer multiple of the frequency resolution (i.e., spectral leakage). The above two issues are addressed by setting up a window function, and since the selected frequencies are far enough apart from each other and the peak amplitudes are the quantity of greater interest, the flat-top window is chosen to be applied to the sample data. It is able to focus the energy on the main lobe as much as possible, with a certain loss of frequency resolution, so that the spectral leakage is effectively limited.

### 3.4. Height-Independent Positioning

We aim to solve the issues mentioned in Section 1 and Section 2 and propose this method based on a trade-off between hardware and computational complexity constraints. It can be derived from (2) that the RSSR between the *i*-th and *j*-th LEDs is

(7) $$\begin{array}{c} \begin{array}{c} \tau_{ij}=\frac{P_{i}}{P_{j}}={\left(\frac{d_{j}}{d_{i}}\right)}^{n+3},\left(i=1,\cdots ,M,j=i+1,\cdots ,M\right). \end{array} \end{array}$$

In practical environments, the measured RSSR contains noise $\Delta \tau_{ij}$,

(8) $$\begin{array}{c} \begin{array}{c} {\hat{\tau }}_{ij}=\tau_{ij}+\Delta \tau_{ij}=\frac{\hat{P_{i}}}{\hat{P_{j}}}. \end{array} \end{array}$$

Now, the problem is finding the coordinate u from the measurement ${\hat{\tau }}_{ij}$. Since RSSR varies nonlinearly with distance, to deal with the nonlinear problem, we shall denote $\rho_{ij}=\sqrt[{m}]{\tau_{ij}^{2}}$, where m=n+3, and then (7) can be transformed into

(9) $$\begin{array}{c} \begin{array}{c} d_{j}^{2}=\rho_{ij}d_{i}^{2},\left(i=\arg\max_{i}P_{i},\;j=1,\cdots ,M,\;j\neq i\right). \end{array} \end{array}$$

Further, we express the distance in the form of vectors by $d_{i}^{2}={\left(s_{i}-u\right)}^{T}\left(s_{i}-u\right)+h^{2}$ and (9) can be shown as

(10) $$\begin{array}{c} \begin{array}{c} b_{ij}-a_{ij}^{T}u+u^{T}u=0\;, \end{array} \end{array}$$

where

(11) $$\begin{array}{c} \begin{array}{c} b_{ij}=\frac{s_{j}^{T}s_{j}-\rho_{ij}s_{i}^{T}s_{i}}{1-\rho_{ij}}+h^{2}\;,\;a_{ij}=\frac{2\left(s_{j}-\rho_{ij}s_{i}\right)}{1-\rho_{ij}}\;. \end{array} \end{array}$$

It is important to emphasize that the coordinates u and s in (10) are 2D rather than 3D. The relative height *h* is presented separately. The noise in RSSR is relatively small due to the Lambert order and distance between the transmitter and receiver. Thus, we can expand $\rho_{ij}$ at the measured ${\hat{\tau }}_{ij}$ by Taylor series and retain the first-order term. Substituting the result into (10), after simplification, yields

(12) $$\begin{array}{c} \begin{array}{c} {\hat{b}}_{ij}-{\hat{a}}_{ij}^{T}u+u^{T}u=\left(\alpha_{ij}^{T}u-\beta_{ij}\right)\Delta \tau_{ij}\;, \end{array} \end{array}$$

where

(13) $$\begin{array}{c} \begin{array}{c} {\hat{b}}_{ij}=\frac{s_{j}^{T}s_{j}-{\hat{\rho }}_{ij}s_{i}^{T}s_{i}}{1-{\hat{\rho }}_{ij}}+h^{2}\;,\;\beta_{ij}=\frac{2\tau_{ij}^{\left(\frac{2}{m}-1\right)}}{m{\left(1-\rho_{ij}\right)}^{2}}\left(s_{j}^{T}s_{j}-s_{i}^{T}s_{j}\right)\;, \end{array} \end{array}$$

(14) $$\begin{array}{c} \begin{array}{c} {\hat{a}}_{ij}=\frac{2\left(s_{j}-{\hat{\rho }}_{ij}s_{i}\right)}{1-{\hat{\rho }}_{ij}}\;,\;\alpha_{ij}=\frac{4\tau_{ij}^{\left(\frac{2}{m}-1\right)}}{m{\left(1-{\hat{\rho }}_{ij}\right)}^{2}}\left(s_{j}-s_{i}\right)\;. \end{array} \end{array}$$

Without losing generality, *i* is fixed at 1 so that j=2,⋯,M. By utilizing (13) and (14), all the (10) can be stacked into a matrix as

(15) $$\begin{array}{c} \begin{array}{c} b_{1}-A_{1}\psi =B\Delta \tau \;, \end{array} \end{array}$$

where

(16) $$b_{1}=\left[\begin{array}{c} b_{12}\\ \vdots \\ b_{1M} \end{array}\right],A_{1}=\left[\begin{array}{c} a_{12}\\ \vdots \\ a_{1M} \end{array}\begin{array}{c} -1\\ \vdots \\ -1 \end{array}\right],B=\left[\begin{array}{ccc} \alpha_{12}^{T}u-\beta_{12} & & \\ & \ddots & \\ & & \alpha_{1M}^{T}u-\beta_{1M} \end{array}\right],$$

(17) $$\Delta \tau =\left[\begin{array}{c} \Delta \tau_{12}\\ \vdots \\ \Delta \tau_{1M} \end{array}\right],\psi =\left[\begin{array}{c} u\\ u^{T}u \end{array}\right].$$

Without losing generality, *i* is fixed at 1 so that *j* = 2,⋯,M. The solution can be given by weighted least squares (WLS) as

(18) $$\begin{array}{c} \begin{array}{c} \hat{\psi }={\left(A_{1}^{T}\mathrm{WA}\right)}^{-1}A_{1}^{T}Wb_{1}\;, \end{array} \end{array}$$

where $W={\left(BQB\right)}^{-1}$, $Q=E[\Delta \tau \Delta \tau^{T}]$ is the covariance matrix of RSSR measurements. In practice, Q is unknown. The WLS is only sensitive to the structure of Q, rather than the actual value [30,31]. Since the propagation channels of each LED are similar, an alternative is replacing Q with an identity matrix.

After obtaining the estimated 2D position $\hat{u}=\hat{\psi }$(1:2), we can turn to estimating the height to obtain the 3D coordinate. Since the third element of $\hat{\psi }$ depends on the height, which is unknown, and the estimation from (18) is inaccurate (see Section 3.5), we shall use ${\hat{u}}^{T}\hat{u}$ to replace $\hat{\psi }\left(3\right)$. Denoting $b_{1}=b+1_{L}h^{2}$ and $\tilde{\psi }={\left[{\hat{u}}^{T},{\hat{u}}^{T}\hat{u}\right]}^{T}$ and putting them into (15) yields

(19) $$\begin{array}{c} \begin{array}{c} \left(b-A_{1}\tilde{\psi }\right)+1_{L}h^{2}\approx B\Delta \tau \;, \end{array} \end{array}$$

where *L* is the number of combinations of RSSR and $1_{L}$ is an all-one column vector of length *L*. Thus, the solution of *h* in terms of WLS is

(20) $$\begin{array}{c} \begin{array}{c} \hat{h}=\sqrt{-{\left(1_{L}^{T}W1_{L}\right)}^{-1}1_{L}^{T}W\left(b-A_{1}\tilde{\psi }\right)}\;. \end{array} \end{array}$$

The net effect of using such a W is the reduction in the covariance matrix of the estimate for ψ [32]. Therefore, we can obtain higher accuracy in receiver position estimation.

### 3.5. Proof of Height Independence

It appears that the above RSSR-based WLS method requires the parameter *h*, and, more importantly, $u^{T}$ and $u^{T}u$ in ψ are coupled. However, in fact, the results obtained by the WLS approach are independent of the height, and we prove this feature mathematically. Consider the solution without weights

(21) $$\begin{array}{c} \begin{array}{c} \hat{\psi }={\left({A_{1}}^{T}A_{1}\right)}^{-1}{A_{1}}^{T}b_{1}=Hb_{1}\;, \end{array} \end{array}$$

where

(22) $$\begin{array}{c} \begin{array}{c} H=({A_{1}}^{T}A_{1}{)}^{-1}{A_{1}}^{T}. \end{array} \end{array}$$

To extract parameter *h*, we apply

(23) $$b_{1}=\left[\begin{array}{cc} b & 1_{L} \end{array}\right]{\left[\begin{array}{cc} 1 & h^{2} \end{array}\right]}^{T}$$

Then, (21) is developed as

(24) $$\begin{array}{c} \begin{array}{c} \begin{array}{cc} Hb_{1}\; & =\left[\begin{array}{c} H\left(1:2,:\right)b+H\left(1:2,:\right)1_{L}h^{2}\\ H\left(3,:\right)b+H\left(3,:\right)1_{L}h^{2} \end{array}\right]. \end{array} \end{array} \end{array}$$

It can be seen from (24) that the estimated coordinate $\hat{u}$ contains the item H(1:2,:)$1_{L}h^{2}$ that is relative to *h*. In order to further simplify H(1:2,:)$1_{L}$, we define $A_{1}=\left[\begin{array}{cc} A & -1_{L} \end{array}\right]$. By exploiting the block matrix inversion to H, we arrive at

(25) $$\begin{array}{c} \begin{array}{c} \begin{array}{cc} H\left(1:2,:\right)1_{L} & ={\left(A^{T}A\right)}^{-1}\left[A^{T}+kA^{T}1_{L}{1_{L}}^{T}A{\left(A^{T}A\right)}^{-1}A^{T}-kA^{T}1_{L}{1_{L}}^{T}\right]1_{L}\\ \; & ={\left(A^{T}A\right)}^{-1}A^{T}1_{L}\left\{1+k\left[{1_{L}}^{T}A{\left(A^{T}A\right)}^{-1}A^{T}1_{L}-{1_{L}}^{T}1_{L}\right]\right\}\;, \end{array} \end{array} \end{array}$$

where

(26) $$\begin{array}{c} \begin{array}{c} k=-{\left[{1_{L}}^{T}A{\left(A^{T}A\right)}^{-1}A^{T}1_{L}-{1_{L}}^{T}1_{L}\right]}^{-1}. \end{array} \end{array}$$

Finally, by substituting (26) into (25), we have

(27) $$\begin{array}{c} \begin{array}{c} H\left(1:2,:\right)1_{L}={\left(A^{T}A\right)}^{-1}A^{T}1_{L}\left[1+\left(-1\right)\right]=0\;. \end{array} \end{array}$$

The result above shows that the final solved $\hat{u}$ are constant regardless of *h*, while the error caused by the height only affects the redundant term $\hat{\psi }\left(3\right)$ instead of $\hat{u}$ according to (24) and (27). After obtaining the 2D coordinate $\hat{u}$, the third element of $\hat{\psi }$ can be replaced by ${\hat{u}}^{T}\hat{u}$ so that the error is reasonable, which means that $\hat{h}$ also can be estimated. In the following experiments, we use the experimental results for verification.

## 4. Experiment and Results

### 4.1. Experimental Setup

**Figure 4:**
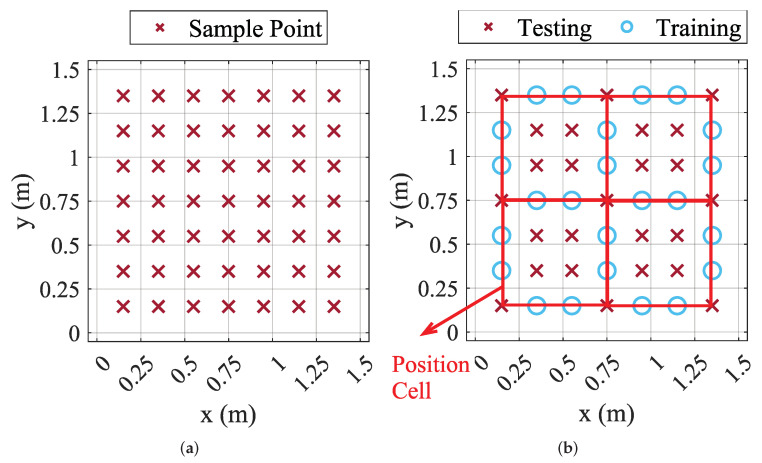
The sample points of each surface. (**a**) The original, and (**b**) the division of ML method.

The experimental setup of the proposed VLP system is shown in Figure 3. The size of our experimental area is 1.5 m × 1.5 m × 2.7 m, where 5 LED lights with a measured Lambert order of 2.51 are distributed at the height of 2.7 m with horizontal coordinates of (0, 0), (0, 1.5), (1.5, 0), (1.5, 1.5), and (0.75, 0.75) in meters, respectively. As detailed in Section 3.2 and Section 3.3, by controlling the MOSFETs (SI2310), the GD32F330 microcontrollers modulate the converted DC into square-wave signals of different frequencies, and then drive the LEDs emitting modulated lights. Each LED light (HGC-T003) has a reflector with a tilt angle of 26 degrees and runs at the same power. As for the receiver side, the current signal converted from PD (SGPN88MQ) is amplified to the voltage signal by a two-stage circuit consisting of a transimpedance amplifier and a voltage amplifier based on the OPA2320, and then the voltage signal is sampled by the built-in ADC of the GD32F330 microcontroller. The microcontroller’s sample rate and sample number are set as 512ksps and 2048. After applying the FFT to the sampled signal, the four most significant RSS values are selected to estimate the horizontal coordinate and the height of the receiver. The positioning error is defined as the Euclidean distance between the estimated and the actual position. The distribution of sample points is shown in Figure 4a. In particular, 49 sampling points (7×7) are selected in the 1.5m×1.5m square area of 0 cm, 20 cm, and 40 cm above the ground, respectively, with each point spaced 20 cm apart. The receiver samples 300 times at each position. Then, it will be moved to the next point manually, until finishing all 49 test points.

To evaluate the 3D positioning performance with a limited number of parameters, the proposed method is compared with the 3D VLP methods, SLLS [29], and the polynomial trilateral ML [22]. Moreover, to evaluate the horizontal positioning performance, we additionally consider a trilateration method designed for the 2D case, which takes the accurate height and the LED emission power as a priori knowledge. The orientation ($u_{R}$) of the PD in SLLS is fixed at ${[0,0,1]}^{T}$ and the convergence threshold is set as $1\times 10^{-4}$. Since only the four largest RSS values are used in the experiment, each sampling plane for the ML-based method is divided into four small squares as positioning cells according to the combination of selected LEDs, as shown in Figure 4b. The 25 points on the diagonal of each positioning cell are used for testing, and the remaining 24 points are used for training.

### 4.2. Horizontal Positioning 2D Performance

**Figure 5:**
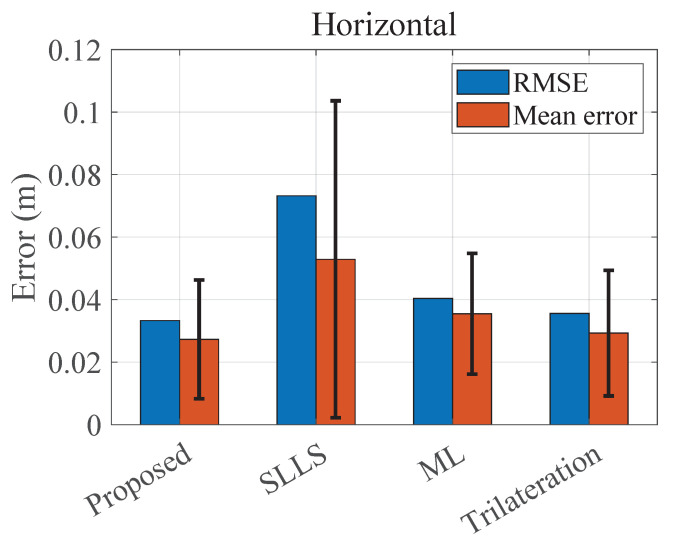
The overall horizontal positioning result; error bars donate S.D.

Figure 5 shows the mean horizontal positioning error of the four methods considered in Section 4.1 for all the positioning points on three test planes, where the relative heights are 2.3 m, 2.5 m, and 2.7 m. The mean horizontal error of the proposed method is 2.73 cm, while the other three methods have the mean error of 5.29 cm, 3.55 cm, and 2.93 cm, respectively.

**Figure 6:**
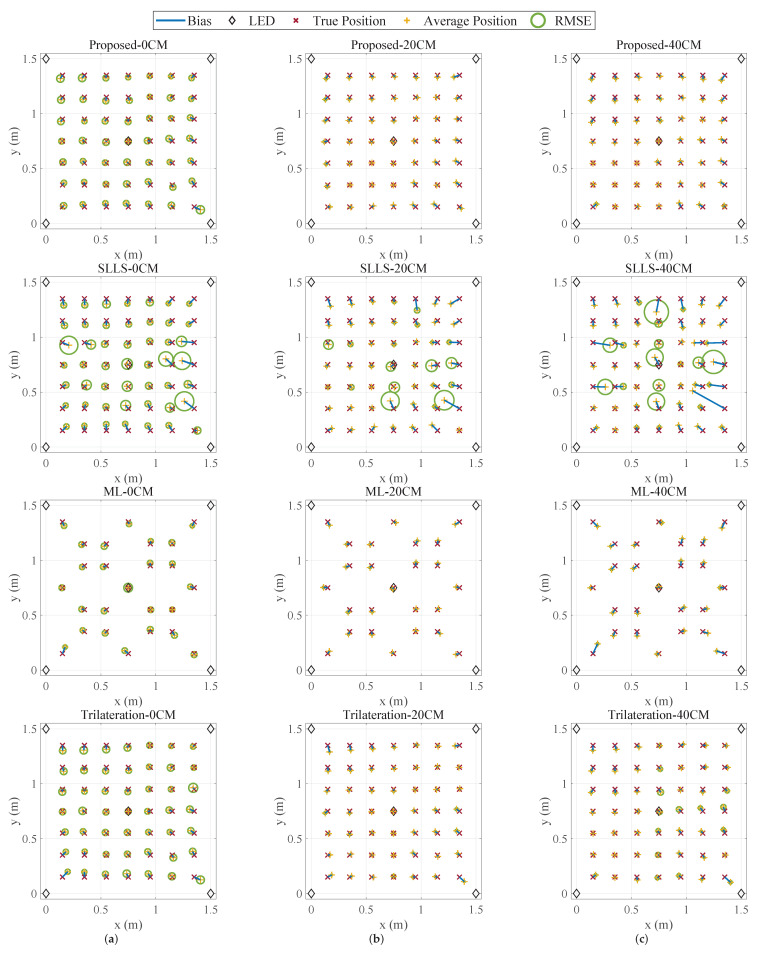
The 2D positioning distribution at different heights: (**a**) 0 cm, (**b**) 20 cm, (**c**) 40 cm.

Figure 6 shows the horizontal positioning distribution at different heights. When the receiver height changes, the positioning accuracy varies as well. It decreases because the signal-to-noise ratio (SNR) is low as *h* is large and the receiver is far from the transmitters, and it also degrades when the receiver is close to the transmitters because the tilted reflectors of the LEDs reflect more NLoS signals and block part of the emitting LoS signal. Furthermore, the positioning result has a bias of 2–6 cm due to system errors in the experimental prototype, such as anisotropy, inconsistency of the LEDs, and the radiation angle of the transmitter being not exactly equivalent to the incident angle of the receiver.

**Figure 7:**
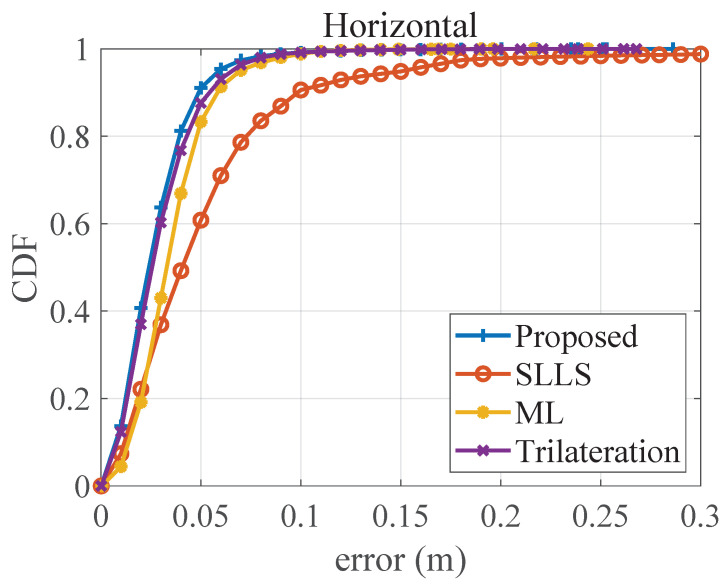
The CDF of horizontal positioning error.

The comparison of these four methods in Figure 6 shows that the proposed method has better horizontal accuracy in the case of lacking a priori height. It has a lower deterioration in the accuracy at the edges of the positioning area. Moreover, the comparison of the proposed method and the 2D trilateration method with accurate height shows that the proposed method is able to achieve similar accuracy despite the fact that the height and emission power are unknown. Figure 7 shows the cumulative distribution function (CDF) of the horizontal positioning errors. In total, 90% of the errors of the proposed method are below 4.8 cm, which is better than the ML-based method (5.8 cm) and the trilateration method (5.4 cm). Moreover, the proposed method requires less a priori information and no offline data collection, making it more operable in practical application scenarios.

### 4.3. Height Independence Verification

**Figure 8:**
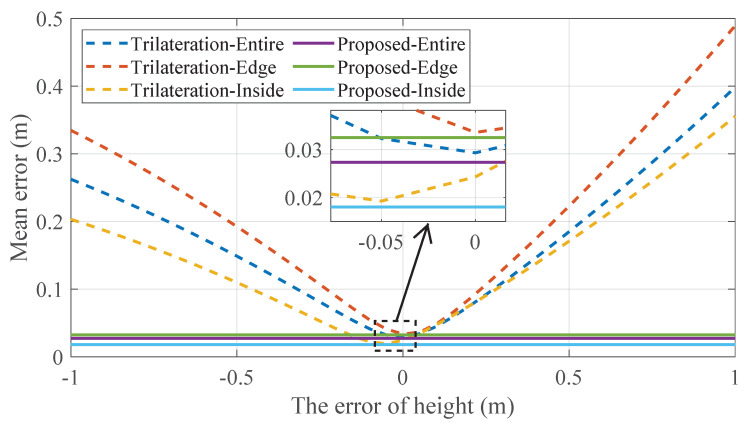
Horizontal positioning error at different a priori heights.

We next evaluate the influence of inaccurate a priori height on the horizontal localization performance. Since the SLLS and ML methods do not require a priori height information, we compare the proposed method with the trilateration method designed for 2D and needing the receiver height. Figure 8 shows the variation in the horizontal positioning error when the height is inaccurate. For the actual relative heights, we perform changes in steps of 0.05 m in the range of [−1m,1m] and calculate the mean error.

The points inside (5 × 5) and the 24 points at the edges are evaluated separately. Consistent with Section 3.5, the positioning results of the proposed method do not change, no matter how much error is included in the relative height. The fluctuation of the mean error is less than $1\times 10^{-14}$ m, which is caused by the calculation accuracy. As for the trilateration position, its minimum mean positioning errors are still slightly higher than the proposed method. Trilateration inside reaches its minimum at an error of −0.05 m, and one possible conjecture is that the error in height compensates for its bias. The entire positioning error increases with the height error, which means that it is vulnerable to height inaccuracy. When the height error exceeds 23.44 cm, the edge positioning accuracy drops to the decimeter level, and the entire positioning accuracy drops to the decimeter level as well when the height error exceeds 27.26 cm. Considering the scene size of this experiment, the decimeter-level error occurs when the height error exceeds 9–12% of the real relative height. This case perhaps arises if the receiver is moved manually and its height changes, where the previously measured height is still used for the trilateration.

### 4.4. 3D Positioning Performance

**Figure 9:**
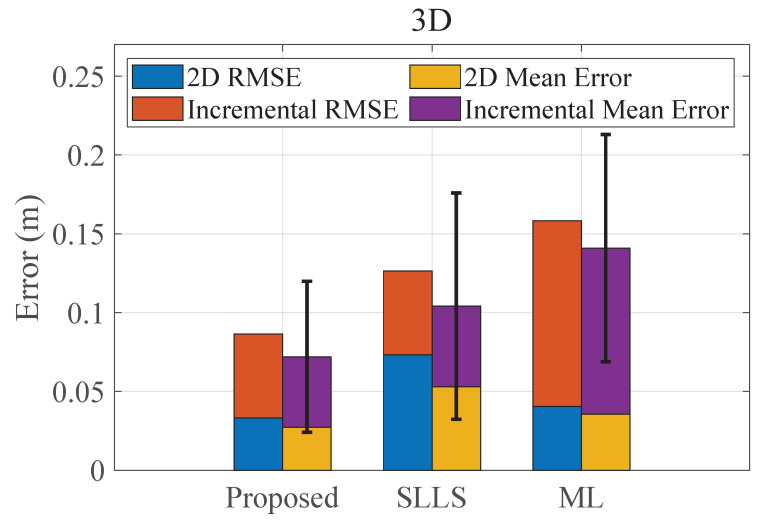
The overall 3D positioning result; error bars donate S.D.

The overall 3D positioning results are shown in Figure 9, and the 3D mean positioning error is expressed by stacking the 2D mean error and the incremental error from the height estimation. The mean error of the 3D positioning for the proposed method is 7.20 cm, while the mean errors of the SLLS-based and the ML-based methods are 12.64 cm and 15.83 cm, respectively. It can be found from Figure 9 that the 3D positioning accuracy deteriorates significantly compared to the 2D accuracy. A common deployment of a VLP system is that the transmitters are all on one side of the receiver and their heights are the same, which makes the vertical dilution of precision (VDOP) larger than the horizontal dilution of precision (HDOP), resulting in the fact that the vertical positioning error is larger than the horizontal positioning error [33].

**Figure 10:**
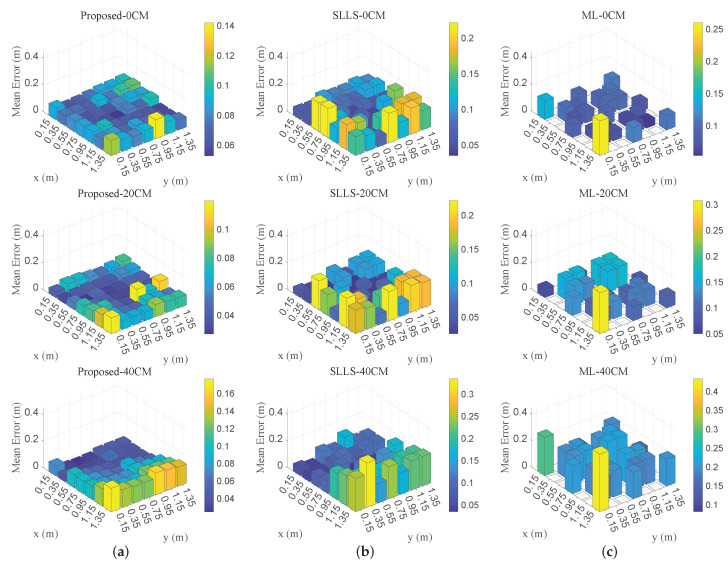
The 3D positioning distribution at different heights: (**a**) the proposed method, (**b**) the SLLS-based method, (**c**) the ML-based method.

**Figure 11:**
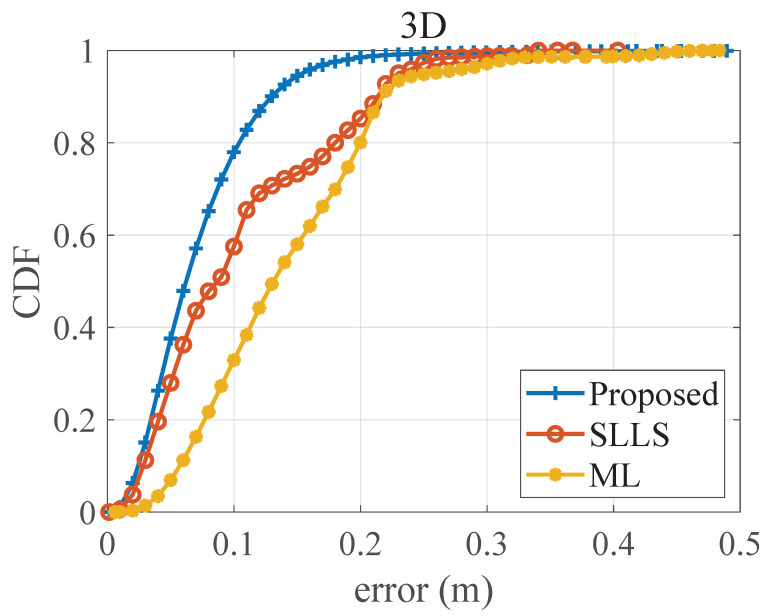
The CDF of 3D positioning error.

Figure 10 shows the 3D positioning error distribution of test planes at different heights. The error of the SLLS method becomes unstable at the edges of the localization area. The error of the ML method is significantly higher in the vertical direction than in the horizontal direction, which may be due to the fact that the regression equation in the ML method cannot provide a good fit for the vertical position estimate. Compared with the SLLS method and the ML method, the proposed method has more accurate positioning performance and most of its errors are concentrated at a lower level. As the CDF curves in Figure 11 show, 90% of the errors of the proposed method are within 13.0 cm. Nonetheless, the SLLS method is less stable where the error curve deteriorates at 0.1–0.2 m, resulting in 90% of errors reaching 21.7 cm, which is close to the ML method.

The above experiments show that the proposed method is able to maintain the positioning accuracy for height without losing the accuracy of horizontal positioning. It can be used in industrial scenarios with automated mobile robots or vehicles, enabling them to position themselves in the event of changes in floor, ceiling, or their own height.

## 5. Conclusions

To develop the VLP technology into practical applications, a height-independent positioning scheme has been proposed in this paper. Essentially, the proposed method leverages the received RSSR from multiple LED transmitters rather than conventional RSS to locate the horizontal coordinates of the receiver. It estimates the horizontal coordinate of the receiver first and can further obtain the height of the receiver to achieve 3D positioning. This new method relaxes the requirements of a priori information such as height and LED emission power, required in traditional positioning techniques. We provide a theoretical analysis of height independence in mathematics. It reveals that a priori height information is not necessary for the novel RSSR-based positioning method, which has been validated by experiments. We build a prototype using low-cost LED luminaires and a microcontroller-based receiver to evaluate the performance. Experimental results show that the proposed method can provide the mean error of 2.73 cm in horizontal coordinate estimation and 7.20 cm in 3D positioning. The proposed method is simple and accurate, and it has the potential for indoor positioning applications with low-cost devices.

## Data Availability

Not applicable.

## Abbreviations

The following abbreviations are used in this manuscript:

VLP	Visible Light Positioning
RSS	Received Signal Strength
3D	Three-Dimensional
2D	Two-Dimensional
VLC	Visible Light Communication
LED	Light Emitting Diode
IoT	Internet of Things
RF	Radio Frequency
PD	Photodiodes
CMOS	Complementary Metal Oxide Semiconductor
TOA	Time of Arrival
TDOA	Time Difference of Arrival
AOA	Angle of Arrival
CSI	Channel State Information
LoS	Line of Sight
ML	Machine Learning
NN	Neural Network
SLLS	Successive Linear Least Squares
AC	Alternating Current
DC	Direct Current
PWM	Pulse Width Modulation
MOSFET	Metal-Oxide-Semiconductor Field-Effect Transistor
ADC	Analog-to-Digital Converter
FFT	Fast Fourier transform
SNR	Signal-to-Noise Ratio
NLoS	Non-Line-of-Sight
CDF	Cumulative Distribution Function
VDOP	Vertical Dilution of Precision
HDOP	Horizontal Dilution of Precision
